# Structural aspects of chemical modifications in the MHC-restricted immunopeptidome; Implications for immune recognition

**DOI:** 10.3389/fchem.2022.861609

**Published:** 2022-08-09

**Authors:** Tatyana Sandalova, Benedetta Maria Sala, Adnane Achour

**Affiliations:** ^1^ Science for Life Laboratory, Department of Medicine Solna, Karolinska Institutet, Stockholm, Sweden; ^2^ Section for Infectious Diseases, Karolinska University Hospital, Stockholm, Sweden

**Keywords:** major histocompatibility complex (MHC), epitope, post-translation modification, T cell receptor (TCR), human leucocyte antigens (HLA), immune responses, immunopeptidome

## Abstract

Significant advances in mass-spectroscopy (MS) have made it possible to investigate the cellular immunopeptidome, a large collection of MHC-associated epitopes presented on the surface of healthy, stressed and infected cells. These approaches have hitherto allowed the unambiguous identification of large cohorts of epitope sequences that are restricted to specific MHC class I and II molecules, enhancing our understanding of the quantities, qualities and origins of these peptide populations. Most importantly these analyses provide essential information about the immunopeptidome in responses to pathogens, autoimmunity and cancer, and will hopefully allow for future tailored individual therapies. Protein post-translational modifications (PTM) play a key role in cellular functions, and are essential for both maintaining cellular homeostasis and increasing the diversity of the proteome. A significant proportion of proteins is post-translationally modified, and thus a deeper understanding of the importance of PTM epitopes in immunopeptidomes is essential for a thorough and stringent understanding of these peptide populations. The aim of the present review is to provide a structural insight into the impact of PTM peptides on stability of MHC/peptide complexes, and how these may alter/modulate immune responses.

## 1 Introduction

**FIGURE 1:**
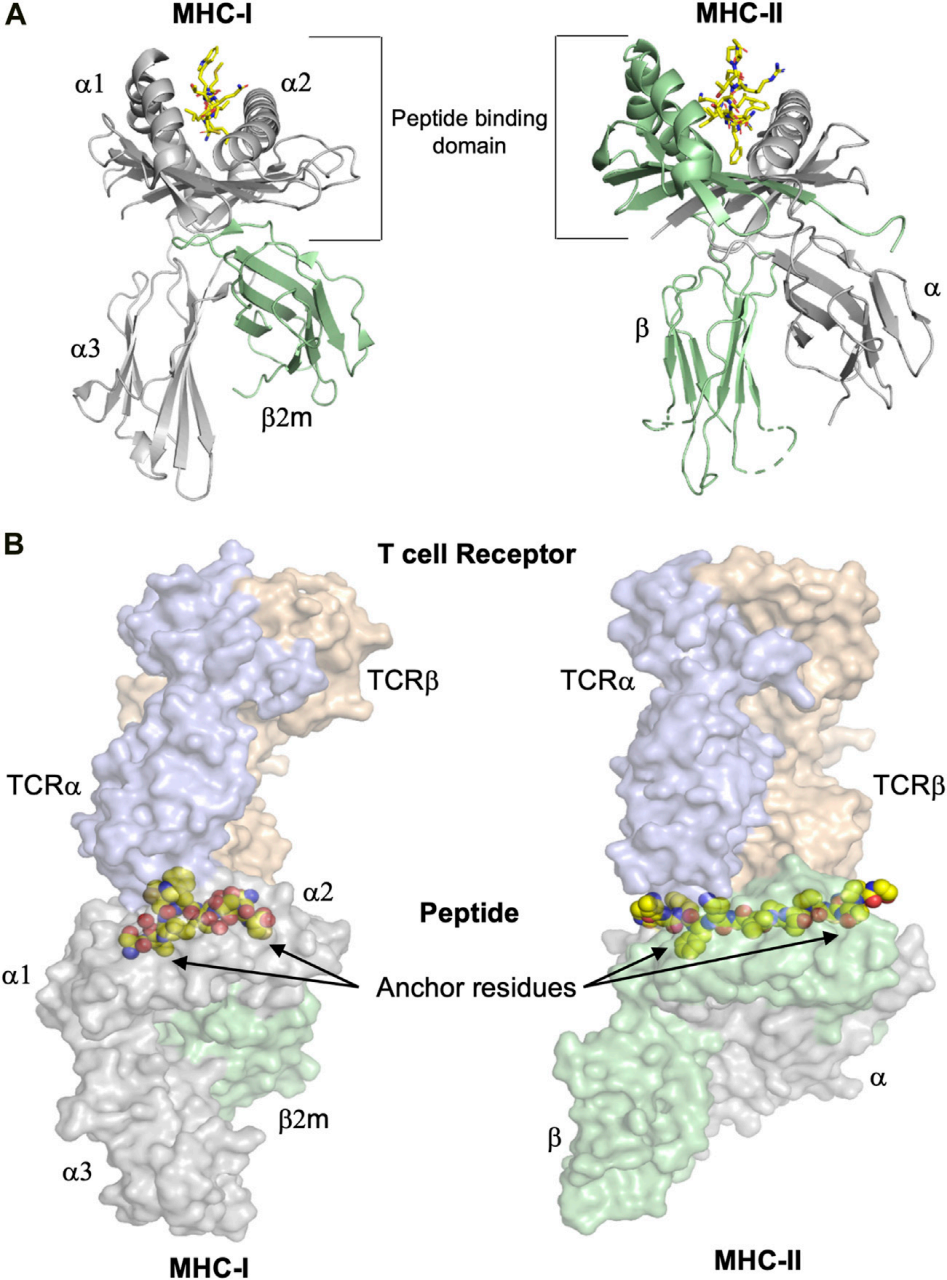
The overall structures of MHC-I and MHC-II complexes are similar. **(A)** Despite the similarity of their overall three dimensional folds, there are some significant differences between MHC-I and MHC-II molecules. Left panel: The polymorphic MHC-I heavy chain, colored in grey, comprises the peptide binding domain (PBD or α1-α2 domain) and an additional Ig-like domain denominated α3. Furthermore, the almost invariable Ig-like β_2_-microglobulin (β2m) domain, colored in green, binds non-covalently between the α1-α2 and α3 entities, stabilizing the overall structure of MHC-I/peptide complexes (pMHC). The peptide is colored in yellow. Right panel: MHC-II molecules consist of the two polymorphic α and β chains (in grey and green, respectively), each one comprising half of the PBD and each one with an additional Ig domain that separates the PBD from the cell membrane. The peptide is colored in yellow. **(B)** T cell receptors bind on the top of pMHC molecules, interacting with both the presented peptides and the MHC chains. The surfaces of both TCR and pMHC are semitransparent. Left panel: The α and β chains of the MHC-I-restricted TCR are colored light blue and orange, respectively, while the surfaces of the heavy chain and β_2_m are in light violet and light green, respectively. The side chains of the presented peptide are represented by yellow spheres. Nitrogen and oxygen atoms are in blue and red, respectively. Classical examples of canonical peptide anchor residues are indicated by arrows, with their side chains buried within specific MHC pockets. The N- and C-termini of MHC-I peptide-binding clefts are closed, restricting most often the length of the bound peptides to 8–10 amino acids. Right panel: The α and β chains of the MHC-II-restricted TCR are in light blue and orange, respectively. The three dimensional structures of the ternary complexes derveal that MHC-II-restricted TCRs most often bind very similarly to their cognate pMHC-II complexes compared to MHC-I-restricted TCRs. In contrast to a majority of MHC-I-restricted peptides, the flanking residues of most MHC-II-restricted epitopes are protruding our from both the N- and C-termini of the MHC-II cleft and are more solvent accessible. All figures were created using PyMOL (The PyMOL Molecular Graphics System, Version 2.3.0, Schrödinger, LLC). All figures were created using the TCR/pMHC ternary structures determined in 2BNR.pdb and 1ZGL.pdb.

The immune system makes use of leukocytes to scan all tissues of the organism for infected and/or stressed cells, followed by their potential elimination. To make this possible, Nature has invented a mechanism based on cell surface presentation of intracellularly processed peptides bound to major histocompatibility complexes (MHC) (Klein 1986). The two classes of MHC molecules, class I (MHC-I) and class II (MHC-II) bind repertoires of endogenously processed peptide antigens, termed immunopeptidomes (Istrail et al., 2004), that are displayed on the cell surface enabling recognition by CD8^+^ cytotoxic T cell lymphocytes (CTL), CD4^+^ T helper cells and NK cells. The overall 3D structures of the extracellular regions of MHC-I and MHC-II molecules are similar, comprising a peptide-binding domain (PBD) and two immunoglobulin-like (Ig) domains that separate the PBD from the membrane (Figure 1A). The peculiar structure of the PBD resembles a cradle with its base formed by eight β-strands and its sides formed by two broken α-helices. The processed peptide is presented on the base of this cradle and is partially entwined between the two α-helices. T cell and most NK cell receptors bind on the top of the pMHC, interacting almost always with both the MHC chains and the presented peptides (Figure 1B).

This review will mainly focus on structural descriptions of the effects of post-translational modifications (PTM) and chemical modifications in MHC-restricted peptides. Thus, although we provide here below a short description of MHC-I and MHC-II antigen processing and presentation, we refer the readers to other more comprehensive and detailed reviews of these pathways (Rock et al., 2016; Dersh et al., 2021). Proteolysis is the main step in intracellular production of peptides that will be loaded onto MHC molecules (Kloetzel 2001). Most MHC-I epitopes usually originate from proteasomal degradation of cytosolic proteins (Blum et al., 2013). Intracellular proteins (including pathogen-associated products) are labeled with ubiquitin and degraded by cytosolic proteasome and/or immunoproteasome. The products are thereafter transferred by the transporter associated with antigen processing (TAP) transmembrane proteins into the endoplasmic reticulum (ER), where an array of peptides is selected and tested for binding to nascent MHC-I by the peptide loading complex (PLC), that includes tapasin, calreticulin and ERp57 (Blees et al., 2017). Finally, the N-termini of peptides are trimmed by ERAP aminopeptidases before and after loading into MHC-I (Saveanu et al., 2005; Kanaseki et al., 2006), and the formed pMHC complexes are thereafter transported via the Golgi system to the cell surface where they are exposed to immune cells. It should be noted that besides the conventional MHC-I antigen processing and presentation pathway described here above, in which peptides result from the degradation of elderly proteins (retirees), several studies have demonstrated that a substantial fraction of peptides can be generated significantly more rapidly, and independently from ubiquitylation or proteasome cleavage, including non-canonical translation of defective ribosomal products (DriPs) or short-lived proteins (SLiPs) (Bourdetsky et al., 2014; Dersh et al., 2021).

Similarly to MHC-I epitopes, a thorough understanding of the molecular processes underlying MHC-II antigen processing and presentation, combined with the discovery of *e.g.* MHC-II-restricted cancer- or other disease-associated epitopes (Linnemann et al., 2015; Alspach et al., 2019), enhances significantly our capacity to design novel and/or improve already existing CD8^+^ and CD4^+^ T cell-based therapies through specific modifications (Chen et al., 2005; van Stipdonk et al., 2009; Duru et al., 2020) In contrast to MHC-I antigen processing, most MHC-II-bound peptides are derived from the endosomal proteolytic machinery. It has been previously thought that a majority of proteins from which MHC-II-restricted peptides are derived are extracellular or host membrane proteins. Indeed, cells endocytose extracellular material as well as membrane proteins, and degrade these after fusion of vesicles with lysosomes. However, it should be noted that mass-spectrometry (MS) studies of MHC-II immunopeptidomes revealed that 25–55% of presented peptides are derived from cytosolic or nuclear proteins, demonstrating that MHC-II antigen processing and presentation covers all kinds of epitopes, similarly to MHC-I molecules (Mommen et al., 2016; Stern and Santambrogio, 2016). The main mechanism providing these intracellular proteins into lysosomes is autophagy, in which protein aggregates or even whole organelles are enclosed by a double membrane forming autophagosomes that fuse with lysosomes, ensuring MHC-II presentation of intracellular protein-derived peptides (Stern and Santambrogio, 2016).

The size of MHC-I and MHC-II binding grooves allows both these molecules to bind 8–10 amino acid (aa) long epitopes in an extended conformation. Most often, peptide binding to different MHC alleles requires the use of two to three peptide anchor residues, with their side chains fitting snuggly within pockets of the MHC peptide binding cleft. There are however specific differences in how these two MHC classes bind peptides. The peptide-binding cleft is closed at both termini by large heavy chain residues that are conserved among most known MHC-I alleles, and that form hydrogen bonds with peptide termini (Figure 1B). These structural restrictions explain why the MHC-I immunopeptidome is significantly enriched with mostly 8–12 aa-long peptides (Caron et al., 2015; Schuster et al., 2018; Yi et al., 2021). However, it should be noted that significantly longer peptides are also always present in the analyzed immunopeptidomes, although in smaller amounts. The closed ends of MHC-I binding clefts do not entirely prevent the extension of peptides at both termini, or only at one end, a feature that seems to prevail more in specific MHC-I alleles compared to others (Mommen et al., 2014). In contrast to MHC-I, MHC-II peptide-binding grooves are open at both ends, allowing presented epitopes to extend out of the cleft (Figure 1B). Accordingly, the MHC-II immunopeptidome is mainly composed of 10–25 aa-long peptides (Sofron et al., 2016; Fugmann et al., 2017; Yi et al., 2021).

The significantly enhanced quality and analytical stringency in sample preparation, MS methods and bioinformatics allow a thorough investigation of the cellular immunopeptidome (Shao et al., 2018; Vizcaíno et al., 2020), revealing that the abundance of each identified peptide ranges from 1 to approximately 10,000 copies per given cell, but also that immunopeptidomes are plastic and molded by cell-intrinsic and -extrinsic factors (Granados et al., 2015)**.** Over the last 15 years, MS methods have allowed the identification and quantification of MHC-restricted peptide populations in a large ensemble of cell lines and tissues. The results of most of these studies are presented in databases such as SysteMHC (https://systemhcatlas.org), a catalog of MHC-I epitopes generated by MS, standardized MHC allele-specific peptide spectral libraries and links to other proteomics and immunology databases (Shao et al., 2018), or caAtlas (http://www.zhang-lab.org/caatlas/) which combines 43 immunopeptidomes containing information from 311 cancer samples from nine different kinds of cancer and over 700 non-cancerous samples (Yi et al., 2021). These MS-based workflows, combined with more reliable MHC ligand prediction algorithms, were particularly helpful to better understand the molecular mechanisms that process cellular and/or phagocyted proteins into epitopes, and identify disease-associated peptides (Mommen et al., 2014; Bassani-Sternberg et al., 2015; Caron et al., 2015; Bassani-Sternberg et al., 2016; Mommen et al., 2016; Pearson et al., 2016; Alpízar et al., 2017; Illing et al., 2018). Besides numbers and sequence identities, these MS studies also provide novel insights into the protein origin of identified peptides, and quantification of peptide amounts that may represent one single protein. For example, more than 12,000 unique peptides were identified in a B-lymphoblastoid cell line, originating from 5,603 proteins located in the ER, nucleus and cytoplasm. The identified immunopeptidome corresponded to more than half of the proteome, composed of about 10,000 proteins. While half of the source proteins were represented by only one single peptide, the remaining proteins were represented by multiple unique peptides, each one binding to a different allele (Mommen et al., 2014). Similarly, a study of 19 different tissues demonstrated the tissue specificity of the immunopeptidome. Among the 28,448 high-confidence H-2D^b^/H-2K^b^-associated peptides, about 30% were detected in only one particular tissue, and a very small amount (0.2% for H-2D^b^ and 1.9% for H-2K^b^) was shared across all 19 studied tissues, emphasizing that the immunopeptidome may be a partial replica of the cellular peptidome (Schuster et al., 2018).

Implementation of different MS workflows also allowed the characterization of large collections of MHC-II-restricted peptides from diseased or healthy tissues. For example, analysis of the immunopeptidome from myelomonocytic leukemia cells expressing HLA-DRB1*10, HLA-DRB1*11 and HLA-DRB3*01 identified 14,000 unique peptides derived from 2,000 intra- and extracellular proteins (Mommen et al., 2016), while a total of 7,400 peptides were characterized in a B-lymphoblastoid cell line expressing different HLA-DQ alleles (Bergseng et al., 2015). The murine immunopeptidome has also been characterized for several cell lines and in connection to different diseases (Sofron et al., 2016; Fugmann et al., 2017). Importantly, analysis of the MHC-II immunopeptidome revealed that, in addition to conventional endosomal MHC-II antigen processing, another previously unknown pathway plays an important role in immune tolerance (Stern and Santambrogio 2016; Unanue et al., 2016). Similar approaches in more recent studies have for example allowed the identification in nine patients of 169 PTM epitopes in a total of 526 unambiguously identified MHC-II-restricted peptides derived from the spike protein of SARS-CoV-2 (Knierman et al., 2020). In conclusion, all these pioneering MS-based studies indicate that the identified immunopeptidomes are robust partial replica of both the intra- and extra-cellular proteome, that can be reliably used for neoantigen discovery, and can be applied to *e.g.* cancer immunotherapy or treatment of autoimmune diseases (Bassani-Sternberg et al., 2016; Wells et al., 2020).

## 2 Post-translational modifications, essential for appropriate cellular functions, can be modulated by pathogen and stress

The connection of the 21 canonical aa in longer chains results in the creation of billions of different proteins, synthetized in different kinds of organisms. Additional functional variety and polymorphisms can be significantly increased through PTM, created either enzymatically or through spontaneous chemical modifications of aa due to environmental modifications. It has been calculated that the combination of all known chemical modifications for aa would multiply their total amount by at least 10 times (Prus et al., 2019). Many PTM are catalyzed by specialized enzymes including kinases, phosphatases, glycosyl- or acyltransferases. PTM have often significant functional effects, increasing protein solubility, modifying protein conformation or affecting protein-protein interactions, which ultimately may alter signaling and modulate intracellular processes. Indeed, some of the most common PTM such as phosphorylation, glycosylation, methylation, acetylation or ubiquitination, are essential for a majority, if not all cellular processes including immune responses (Mann and Jensen 2003; Seo and Lee 2004; Zavala-Cerna et al., 2014). Several key proteins within immunological responses, *e.g.* MHC, T/NK cell receptors and antibodies are commonly glycosylated. Furthermore, all downstream signaling following adequate ligand recognition by a receptor usually starts by phosphorylation of key signaling proteins. While the importance of glycosylation and phosphorylation is thus well-established, more recent studies revealed the significant impact of other kinds of PTM. Acetylation and deacetylation of lysine residues in histones is one of the most well studied epigenetic regulation for gene transcription, with histone acetylation eliciting transcriptional activity (Sterner and Berger 2000). Lactylation of the promoter region in histones links metabolism and gene regulation, as observed in *e.g*. activated macrophages, where histone lactylation increases gene expression associated with wound healing (Zhang D. et al., 2019). Metabolic changes in immune cells are as important for understanding specific immune effector responses as discrete signaling pathways. Lysine residues in non-nuclear proteins can become acetylated, malonylated, succinylated or itaconated by intermediates of the Krebs cycle, which all modify the charge of PTM lysine residues and affect protein-protein interactions (Diskin et al., 2021). For example, malonylation of the mammalian target of rapamycin mTOR modulates its activity (Bruning et al., 2018), while specific succinylation of pyruvate kinase 2 induces its nuclear translocation and increases production of IL-1β (Wang et al., 2017).

PTM are important in viral infections. Host cells make use of specific PTM to reduce viral replication, while viruses use PTM (most often using the host own PTM system) to enhance specific virulence properties and/or escape immune recognition. Altogether, PTM used by viruses or by the host cell to protect itself are fundamental for the outcomes of infections. Besides the well-established effects of ubiquitination and SUMOylation on elimination of viruses (Kumar et al., 2020; Proulx et al., 2020), the importance of several other PTMs in proteins that participate in host-viral interactions have been described (Chang et al., 2016; Chen et al., 2018). In addition to phosphorylation events, lysine (de-)acetylation reorganized the cytoskeleton of HIV-infected cells, and binding of ENV/GP120 to CD4 induces the formation of acetylated α-tubulin which stimulates HIV infection (Chang et al., 2016).

PTMs including phosphorylation, methylation, acetylation and deamidation impact also on cellular DNA sensor functions using diverse mechanisms, such as direct regulation of enzymatic activity, protein-protein and protein-DNA interactions, protein translocations and protein turnover (Song et al., 2021). ADP-ribosylation, which is the addition of one up to 200 ADP-ribose moieties from NAD to Ser, Glu, Asp or Arg residues on intra- or extracellular target proteins regulates T and B cell function and differentiation capacities (Rosado and Pioli 2021). Several bacterial toxins represent significant virulence through their ADP-ribosylation capacity. The *V*. *cholerae* toxin, the heat-labile enterotoxin from *E*. *coli* and the *B. pertussis* toxin are all able to ADP-ribosylate host heterotrimeric G proteins, leading to constitutive activation of adenylate cyclase and increased intracellular concentration of cAMP inducing efflux of water and chloride ions from host enterocytes. It has also been shown that histones, DNA repair proteins, transcription factors and chromatin regulators can also be ADP-ribosylated and that the overall negative charge of (poly)-ADP-ribose strongly affects their functions.

It is also well established that antibody glycosylation is critical for its function (Rudd et al., 2001). For example, the IgG constant domain Fc has a conserved N-glycosylation site at Asn297 which is essential for the recognition of Fc by its receptors (Liu and Liu, 2021). This N-glycan is biantennary and complex, with a core composed of two N-acetylglucoseamine moieties and three mannose molecules. The glycan core is later modified in the Golgi organelle by several glycosyltransferases that add fucose, galactose and sialic acid. Thus, the constant domain Fc is heterogeneous with 36 possible glycan structures due to variations in fucosylation, galactosylation and sialyation (Jennewein and Alter, 2017). Infection can modify the antibody glycosylation pattern such that Fc-glycan patterns in HIV-specific antibodies will be different from HBV- or Dengue-specific antibodies in the same patient (Irvine and Alter, 2020). A large ensemble of PTM play also often a key role in the initiation of autoimmune diseases, including the possible role(s) of pathogens in the provocation of autoimmune responses (Zavala-Cerna et al., 2014). PTM are also emerging markers of aging-related diseases (Santos and Lindner, 2017) and are very important in cancer development. Modulation of metabolism in stressed cells modifies PTM pathways. Indeed, glycans on the surface of a protein can affect both intracellular and extracellular signaling, as well as cell migration and adhesion (Campbell and Wellen 2018; Thomas et al., 2021). Alterations in phosphorylation pathways result in serious outcomes in cancer development and drugs targeting phosphorylation pathways are promising agents in cancer therapy (Singh et al., 2017). Other PTM including arginine methylation or PTM in the extracellular matrix are both correlated with cancer progression (Leeming et al., 2011; Jarrold and Davies, 2019). Increased levels of oxidative stress in cancer cells can lead to protein nitrotyrosination (Ene et al., 2020), or to a shift in the thiol/disulfide equilibrium resulting in cysteinylated or glutathinylated cysteine residues (Yi et al., 2021).

## 3 Major histocompatibility complexes-bound chemically modified peptides exist *in vivo*

Since the immunopeptidome is a robust replica of the proteome, PTM should be found in complex with MHC molecules on the surface of antigen presenting cells, as anticipated in early stages of pMHC studies (Andersen et al., 1999; Haurum et al., 1999). More definitive answers appeared through MS/bioinformatics analyses which revealed that PTM peptides could constitute 12–25% of the immunopeptidome (Zarling et al., 2000; Kastrup et al., 2001; Prus et al., 2019). Indeed, among 12,000 MHC-I-associated peptides identified with high-confidence in a human B-cell line, about 1,500 carried PTM (Mommen et al., 2014). The most common modification was methionine oxidation, found in 988 peptides. Other prominent PTM included 246 peptides with asparagine-cyclization, 196 that were cysteinylated, 83 with deaminated asparagines, 44 with phosphorylated serines and 15 with phosphorylated threonines.

A similar prevalence for PTM was found in the immunopeptidome assessed on C1R cell lines transfected with three of the most common HLA-A allotypes; HLA-A*01:01, HLA-A*02:01 and HLA-A*24:02 (Mei et al., 2020). About 10,000 peptides were detected for each of these transfected MHC-I alleles and an unbiased PTM search revealed that ∼21, 22 and 17% of peptides presented by HLA-A*01:01, HLA-A*02:01 and HLA-A*24:02 carried PTM, respectively. Here again, methionine oxidation accounted for the largest proportion (57–83%) of the identified PTM-epitopes across all HLA allotypes, followed by asparagine/glutamine deamidation (2.5–7%), serine/threonine/tyrosine phosphorylation, while arginine citrullination accounted for less than 3% of the detected PTM repertoire. While all other PTM were relatively much lower in frequency, these infrequently occurring modifications still represented 33% of the detectable PTM space.

Several studies have identified and described MHC-restricted glycosylated peptides. A total of 36 different HLA-B*07:02-restricted O-GlcNAcylated peptides were identified on various primary leukemia samples (Malaker et al., 2017b). Interestingly, these O-GlcNAcylated epitopes displayed different states of glycosylation, comprising in some cases two differently modified residues, thus adding to the polymorphism of the MHC peptide flora. For example, residue p4T was phosphorylated while p7S was glycosylated in the epitope RVKpTPTgSQSY derived from the protein ZNF218 (Marino et al., 2015). Altogether, it is now well established that a large array of chemically modified peptides is presented by MHC-I. The modified residues are most often centrally localized (residues 3–7), and can thus interact with T and/or NK cell receptors, or can be recognized by antibodies.

Post-translational modifications in MHC-II-restricted antigens have attracted a lot of attention due to their involvement in initiation and development of autoimmune diseases or allergies (Zavala-Cerna et al., 2014; Unanue et al., 2016). Studies on rheumatoid arthritis (RA) demonstrated that an ensemble of PTM in self-proteins could be involved in its pathogenesis, exemplified by the key role played by a protruding glycosylation in the immunodominant epitope CII_256-270_ derived from the type II collagen CII protein in the elicitation of autoreactive T cells (Michaëlsson et al., 1994). Studies of self-peptides associated with RA revealed a strong correlation between citrullination of arginine at peptide position p4 and epitope binding affinities to HLA-DRB1 (Scally et al., 2013; Ting et al., 2018; Lim et al., 2021). Others demonstrated that citrullination of several arginine residues in α-enolase-derived peptides elicited significant functional CD4^+^ T cell responses in primary cells from RA patients (Gerstner et al., 2016; Pieper et al., 2018). Similarly, several CD4^+^ T cell clones, identified in studies of allergic responses to bee venom, proliferated in the presence of the glycosylated bee venom phospholipase A2 but not in the presence of free carbohydrate or non-glycosylated protein (Dudler et al., 1995). Several PTM including phosphorylation, methylation and acetylation are frequently found in other autoimmune diseases such as systemic lupus erythematosus (SLE) (Zavala-Cerna et al., 2014). Peptide LL37 derived from the C-terminal part of the human cathelicidin antimicrobial peptide (residues 134–170) acts as a T-cell/B-cell autoantigen in SLE and psoriatic disease (Lande et al., 2021). T-cell epitopes derived from citrullinated LL37 (which comprises six arginine residues) provoke significant CD4^+^ T cell mediated responses in SLE-associated HLA-DRB1*1501/HLA-DRB5*0101 backgrounds compared to unmodified LL37. Interestingly, non-enzymatic carbamylation of lysine side-chains (six residues in LL37) and/or of the N-terminus of the LL37 peptide, appeared to be very important in SLE, since carbamylate-LL37-specific antibodies and CD4^+^ T-cells, both correlated with disease progression, circulate in SLE patients serum (Lande et al., 2021).

A great majority of the hitherto identified MHC-restricted PTM peptides is presented in the database caAtlas (http://www.zhang-lab.org/caatlas/) which comprises 14,172 PTM epitopes with 146 distinct types of modifications (Yi et al., 2021). Analysis of this comprehensive database indicated that: 1) some PTM alter peptide binding to MHC-I, either by changing the position of anchor residues and/or by forming a bulge in the middle part of the PTM peptide. Indeed, although a majority of modified MHC-I antigens have the same length distribution compared to unmodified epitopes, several modifications such as serine, threonine or cysteine acetylation, N-terminus acetylation, methylation of polar residues and arginine/asparagine dimethylation may result in enrichment of longer peptides in the peptidome. While some PTM such as phosphorylation can be found only on solvent-accessible residues, citrullination can occur on both peptide anchor residues or residues that are solvent accessible; 2) The frequency of the same PTM is different in MHC-I- compared to MHC-II- restricted epitopes. Dioxidation is the most abundant modification found in MHC-I- whereas deamidation is most frequent in MHC-II-restricted peptides. MHC-I and MHC-II seem to also have different aa preferences for the same type of PTM. For example, methionine sulfur oxidation is more common in MHC-I antigens, while cysteine sulfur oxidation is more usually found in MHC-II epitopes; 3) MHC-I-restricted antigens are more restrictive in the position of the introduced modifications. Most often phosphorylation occurs at the fourth or eighth residues in MHC-I antigens, while all residues from p1 to p15 can be phosphorylated on MHC-II antigens; 4) Peptide modification ratio is different for distinct PTM. For example, of the 68 unique peptides identified with dioxidated residues, none exists unmodified (Yi et al., 2021). An additional amount of 1,368 peptides were identified both with dioxidated or unmodified residues within the MHC-I immunopeptidome. For MHC-II, only 42 unique peptides were identified with dioxidated residues and 477 other peptides were found both in modified and unmodified states. Thus it is not unusual that only the PTM epitope is processed and presented by MHC.

## 4 Structural insights in major histocompatibility complexes-restricted post-translational modifications peptides

While previous reviews have described the functional effects of PTM T cell epitopes (Kastrup et al., 2001; Petersen et al., 2009b; Unanue et al., 2016), our main aim here is to provide a structural understanding of the effects of PTM on MHC-restricted epitopes and, when possible, describe the molecular bases underlying how these modifications may alter immune responses.

### 4.1 Glycopeptides

Glycosylation is one of the most common PTM (Wolfert and Boons 2013). Indeed, almost all of the key proteins that are involved in the cellular immune system are glycoproteins (Rudd et al., 2001). Several glycosyltransferases and glycosidases catalyze the addition of glycan moieties onto proteins in the ER and Golgi organelles. There are commonly two types of glycans; N-linked glycans with a N-acetylglucosamine sugar moiety bound to asparagines and O-linked glycans with a sugar moiety bound to serine, threonine or modified hydroxylysine residues. Protein glycosylation is very diverse, stretching from only a single sugar in some sites to branched chains composed of several different carbohydrates in others. Although a major function of glycans is to contribute to protein stability and solubility, including appropriate molecular orientation and reduction of unspecific protein-protein interactions, glycosylation in MHC peptides can form neoantigens, and can be used by pathogens to modulate immune responses. As already stated above, all the hitherto described immunopeptidomes include glycosylated peptides (Mommen et al., 2014; Marino et al., 2015; Malaker et al., 2017b). Moreover, T cell responses to MHC-restricted glycoepitopes have been described in tuberculosis (Harriff et al., 2017), for several viruses (Hafstrand et al., 2017; Bagdonaite and Wandall 2018), including HIV (Olvera et al., 2020), and in tumors (Bennun et al., 2013; Peixoto et al., 2019). Reprograming of cancer cells has a strong impact on their glycosylation system, leading not only to overexpression of common glycoproteins, but also to expression of cancer-specific glycoproteins. The functional importance of glycosylated epitopes for T cell elicitation has been previously described (Kastrup et al., 2001; Petersen et al., 2009b; Unanue et al., 2016).

In contrast to N-glycosylated peptides which are not usually present in the MHC-I immunopeptidome, a large amount of peptides with deamidated asparagines (70–200 per 10,000 detected peptides) has been identified (Mommen et al., 2014; Malaker et al., 2017b; Mei et al., 2020). This was actually expected since it is known since the 1990th that mammals possess the cytosolic peptide:N-glycanase that cleaves N-glycoproteins, generating free glycans and deglycosylated peptides in which N-glycosylated asparagines are converted to aspartates (Skipper et al., 1996; Suzuki et al., 2016). Deamidation is the second most common PTM found in the MHC immunopeptidome, comprising 2.5–7% of all detected PTM epitopes (Mei et al., 2020). The most common N-glycosylation motif NX (S/T) is present in 60% of peptides with deamidated asparagines. Indeed, threonines and serines are often found at peptide position +2 after a deamidated asparagine across all MHC-I peptides that have been studied, whereas this has not been observed for glutamine deamidation (Mei et al., 2020). It became therefore clear that most epitopes with deamidated asparagines found in MHC-I immunopeptidomes arise most often from deglycosylated peptides (Mei et al., 2020)*.* To our knowledge, only one study until now has demonstrated the presence of an N-glycan on the MHC-I peptide KPAPPFgNVTV derived from the membrane-bound interleukin 21 receptor (IL21R) (Marino et al., 2015). The same study hypothesized that the glycanase enzyme failed in this specific case to remove the truncated N-glycan moiety.

**FIGURE 2:**
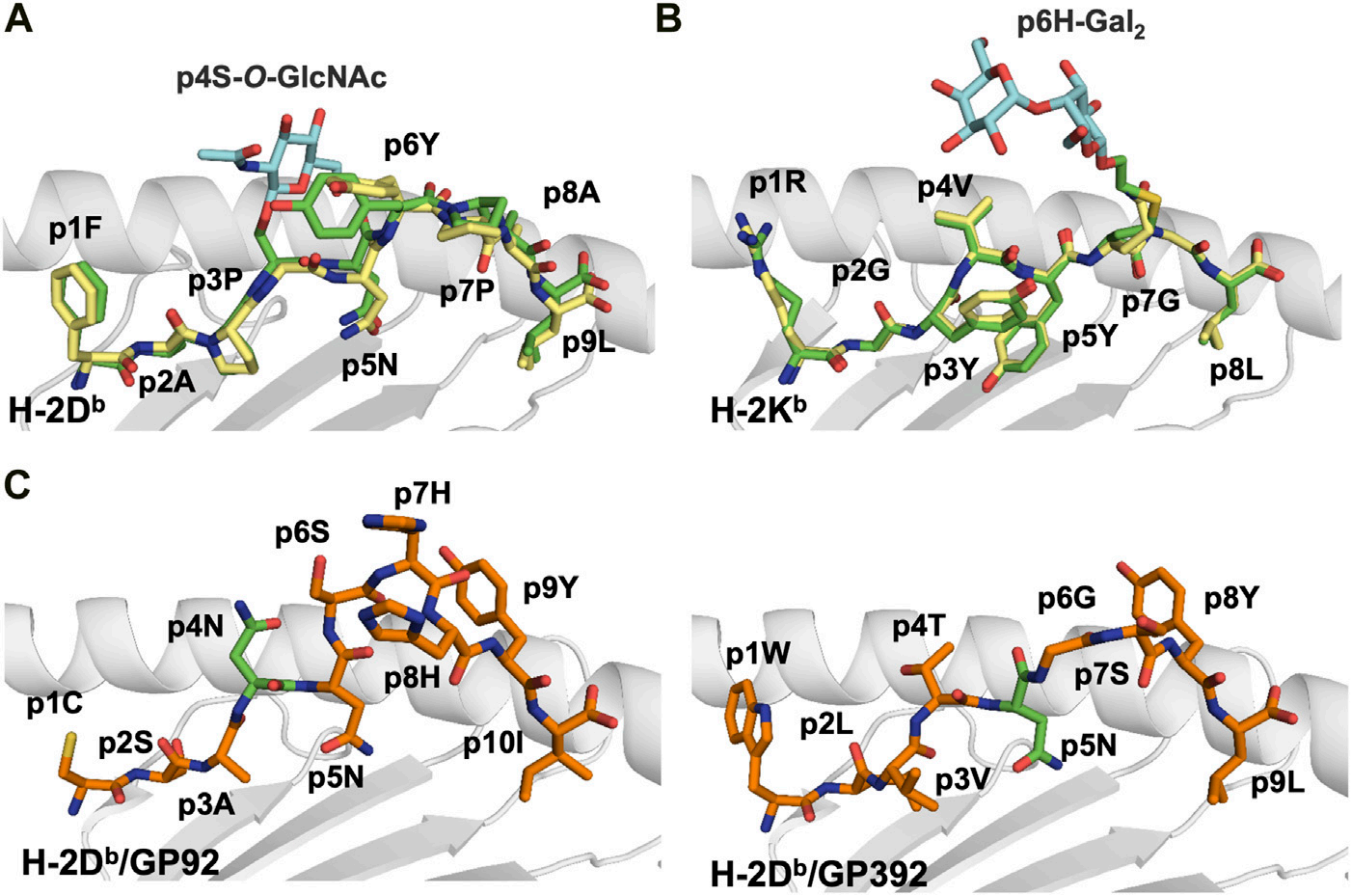
Glycosylation occurs most often in the middle section of the peptide creating a neoantigen that can break tolerance and select for different T cell repertoires **(A)**. Comparative analyses of the crystal structures of H-2D^b^ in complex with the immunodominant Sendai virus epitope FAPGNYPAL (1CE6. pdb) colored in yellow and with the glycopeptide analog K2G with a glycan at p4 (1QLF.pdb), colored in green) (Glithero et al., 1999) reveal the similarity of the peptide backbones. In contrast, the protruding glycan moiety (in blue) may impair/modulate TCR recognition **(B)**. Comparison of the crystal structures of H-2K^b^ in complex with the vesicular stomatitis virus (epitope RGYVYQGL (2VAA.pdb, in yellow) (Fremont et al., 1992) and the glycosylated RGY8-6H-Gal2 (1KBG.pdb, in green) (Speir et al., 1999) demonstrates their high conformational similarities. In contrast, the central region of the putative TCR binding site is dominated by the extensive exposure of the tethered carbohydrate (colored in blue), which forms a neoantigen. **(C)**. Glycosylation of MHC-I-restricted peptides can be used by pathogens to either impair TCR recognition of specific epitopes or reduce the overall stability of pMHC complexes. This is exemplified by the crystal structures of the LCMV epitopes GP92 and GP392 in complex with H-2D^b^. While glycosylation of the asparagine residue at peptide position 4 in GP92 (5JWE.pdb, in green) may impair TCR recognition, glycosylation of the main anchor asparagine residue at p5 in GP392 (5JWD.pdb, in green) may reduce significantly pMHC stability (Hafstrand et al., 2017).

Although N-glycosylated peptides are not usually presented by MHC-I *in vivo*, loading of *in vitro* modified epitopes into MHC-I could be used for immunotherapy. If the glycosylated asparagine does not act as an anchor position, the sugar moiety should not modify the overall conformation of the PTM peptide compared to its wild-type counterpart, as demonstrated in crystal structures of three artificial MHC-I/glycopeptide complexes. Two N-glycosylated PTM peptides were created using the H-2D^b^-restricted Sendai virus nucleoprotein peptide SEV_324–332_ (FAPGNYPAL) as a template. This prototypic high affinity immunodominant peptide binds to H-2D^b^ using the anchor residues p5N and p9L (Fremont et al., 1992). The glycopeptide analogs FAPS(O-GlcNAc)NYPAL (K3G) and FAPGS(O-GlcNAc)YPAL (K2G) carry glycan substitutions at p4 and p5, respectively (Glithero et al., 1999). In both K2G and K3G, the glycan moiety is exposed to the solvent, fully available for direct recognition by TCRs (Figure 2A).

The crystal structure of an artificial glycopeptide variant of the H-2K^b^-restricted vesicular stomatitis virus (VSV) immunodominant peptide (RGYVYQGL) has also been previously determined (Speir et al., 1999). Immunization with the glycopeptide RGYVY(HomoCys-Gal_2_)GL generated polyclonal CTL populations that specifically killed cells presenting the MHC/glycopeptide complex. Comparative analysis of the glycopeptide variant with wild-type VSV (Fremont et al., 1992) demonstrated that the glycan did not affect the conformation of the backbone of the modified peptide nor the overall structure of the H-2K^b^ heavy chain (Figure 2B). Thus, these studies demonstrated that it is possible to design artificial MHC-I-restricted glycosylated neoantigen variants that elicit strong CD8^+^ T cell responses.

Infection of H-2^b^ mice by the lymphocytic choriomeningitis virus (LCMV) generates CD8^+^ cytotoxic T lymphocytes (CTL) directed towards several MHC-I-restricted peptides including the H-2D^b^-restricted minor epitopes GP92 (CSANNSHHYI) and GP392 (WLVTNGSYL), both glycosylated at N-glycosylation sites in full length proteins (Hastie et al., 2016). Interestingly, glycosylation affected differently the affinity of GP92 and GP392 to H-2D^b^, and their immunogenicity. While all three forms of GP92, wild-type (p4N), glycosylated (p4N-glyco) and deamidated (p4D) were immunogenic (Hudrisier et al., 1999; Hudrisier et al., 2001), vaccination with glycosylated GP392 (p5N-glyco) or deamidated GP392 (pD5) were not immunogenic. The crystal structures of H-2D^b^/GP92 and H-2D^b^/GP392 revealed that the observed functional differences correlated with the position of the glycosylated residue. Indeed, while p5N glycosylation impairs binding of GP392 to H-2D^b^, glycosylation of p4N, which protrudes towards the solvent in GP92, possibly allows for immune escape and/or forms a neo-epitope that may select for a different set of CD8^+^ T cells (Figure 2C) (Hafstrand et al., 2017).

Unlike MHC-I, studies of MHC-II immunopeptidome revealed the presence of both N- and O-glycosylated peptides, which is due to the fact that N-glycans are not destroyed in endolysosomes. A total of 93 MHC-II-restricted glycosylated peptides were identified in melanoma cells (Malaker et al., 2017a), including 17 glycoforms composed by six different sugars. Interestingly, a vast majority of the identified epitopes were glycosylated on flanking residues, stretching out from the PBD. Unfortunately, no crystal structure of any MHC-II presenting a glycopeptide has been determined yet. However, three-dimensional molecular models indicated that the glycans could be accommodated in the standard TCR/pMHC geometry (Malaker et al., 2017a). More recently, glycosylated HLA-DR-bound peptides were identified in monocyte-derived dendritic cells pulsed with the SARS-CoV-2 spike (S) protein (Parker et al., 2021). A total of 209 unique peptides derived from the S-protein were identified, many of them displaying glycan moieties. Comparison of the glycosylation profile of the free S protein to that of HLA-II-bound S-derived peptides revealed that several peptide glycans were trimmed, most probably during antigen processing. Thus, the diversity of glycans decorating a specific MHC-restricted peptide may add even more to the overall diversity of the presented immunopeptidome.

### 4.2 Phosphopeptides

Reversible phosphorylation of serine, threonine and tyrosine residues performed by kinases and protein phosphatases plays a critical regulatory role in cells, and its deregulation may be associated with diseases. Malignant transformation modifies often protein kinase/phosphatase pathways which regulate cell growth, proliferation, differentiation and cell death (Du and Lovly 2018; Revathidevi and Munirajan 2019). Fast growing evidence supports the notion that phosphoproteins can be processed intracellularly by usual endogenous pathways which produce complexes of MHC-I and MHC-II- bound modified peptides for specific recognition by CD8^+^ and CD4^+^ T cells, respectively (Zarling et al., 2000; Depontieu et al., 2009). Indeed, phosphopeptides were identified in immunopeptidomes although not in high abundance, usually representing 0.5–1% of detected peptide populations, and 3–4% of all PTM epitopes. Moreover, it has been demonstrated that MHC-I presentation of phosphorylated peptides is TAP-dependent and that the formed pMHC complexes can be recognized by specific T cell receptors (Zarling et al., 2000). Such phosphopeptides could thus be potentially exploited in T-cell-based immunotherapy (Zarling et al., 2000; Zarling et al., 2006; Alpízar et al., 2017; Mei et al., 2020).

Analysis of more than 10,000 HLA-A*0201-restricted peptides from two melanoma cell lines (DM331 and SLM2), an ovarian carcinoma cell line (COV413) and an Epstein-Barr virus-transformed B lymphoblastoid cell line allowed the identification of 36 novel individual phosphopeptides, five of which were restricted to solid tumors and common to both melanoma and ovarian cancers (Zarling et al., 2006). 21/36 were phosphorylated at p4, and a vast majority (18/21) comprised a basic residue at p1. We find this observation interesting since it coincides with the kinase motif RXXS/T. Vicinity of a positively charged residue to the phosphorylated residue is essential not only for kinase binding but also to stabilize pMHC (see below). A total of 150 additional unique MHC-II-restricted phosphopeptides were identified in four cell lines, including two melanoma lines and two autologous EBV-B cells (Depontieu et al., 2009). Phosphate moieties were bound to serines in 93% of identified PTM peptides, while threonines and tyrosines were phosphorylated in 5.3 and 1.7%, respectively. These results coincide roughly with frequencies found in the phosphoproteome of HeLa cells (Olsen et al., 2006). Most identified phosphopeptides were expressed at 6–50 copies per cell. Finally, all these 150 unique PTM peptides were derived from 53 different protein sources from all cellular compartments, and most parent proteins participate in essential cellular pathways (Depontieu et al., 2009).

**FIGURE 3:**
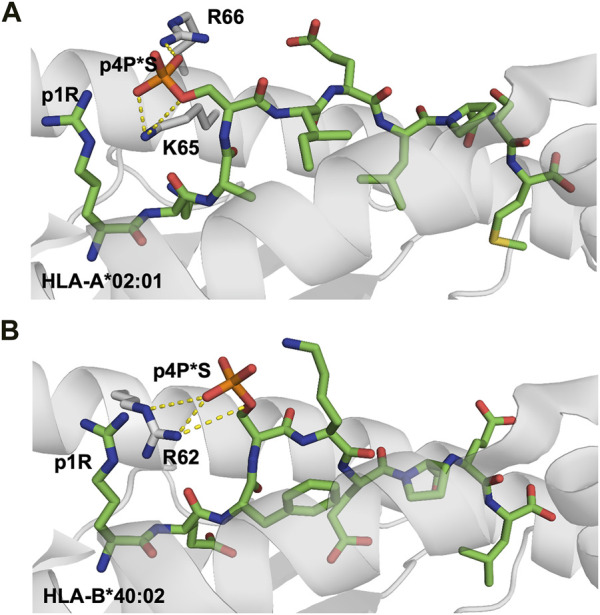
Phosphorylation of MHC-I peptide residues may act as additional external anchor positions, increasing significantly pMHC stability **(A)**. Phosphorylation of the solvent-exposed serine residue at p4 in the HLA-A0201-restricted decameric Lymphocyte specific protein 1-derived peptide (3BH8. pdb) (Mohammed et al., 2008) results in the formation of strong electrostatic interactions between the negatively charged phosphoserine moiety and the surrounding MHC-I residues R66 and K65. Additional interactions are also formed between the phosphorylated moiety and the side chain of peptide residue p1R. Interactions between the phosphate moiety and the basic HLA residues are displayed with yellow dashed lines **(B)**. Similarly, besides forming a hot spot for TCRs, the phosphorylation of the exposed residue in a nonameric peptide derived from the inner centromere protein bound to HLA-B*40:02 (2IEH.pdb) (Alpízar et al., 2017) increases significantly the overall stability of the modified pMHC complexes through the formation of electrostatic interactions with surrounding MHC heavy chain and peptide residues.

A total of 18 crystal structures of HLA-A2 and HLA-B40 in complex with 10 different peptides in phosphorylated and non-phosphorylated forms have been hitherto determined (Mohammed et al., 2008; Petersen et al., 2009b; Alpízar et al., 2017; Mohammed et al., 2017), revealing the structural bases underlying the preferences described above. The location of the phosphate modification was conserved in epitopes with different lengths and different main-chain conformations, with a phosphorylated serine residue at p4. The phosphate moiety forms salt bridges with the two positively charged heavy chain residues Arg65 and Lys66 that border the peptide binding groove of HLA-A2 and the first Arg/Lys residue on the same peptide (Figure 3). Thus, phosphorylation at p4 increases peptide binding affinity for HLA-A2 through elegant use of the solvent-exposed phosphate moiety as an additional ‘anchor’ position although it protrudes towards the solvent. Indeed, phosphorylation at p4 enhanced both peptide binding affinity and overall complex stability by 2 to 160-fold (Mohammed et al., 2008). The importance of residue Arg65 for the enhanced phosphate-induced stabilization was demonstrated using the R65G mutated variant. Removal of the arginine side chain at position 65 reduced significantly the affinity of phosphopeptides, especially in the additional absence of a basic residue at peptide position p1. Interestingly, the combination of the two positively charged residues Arg65 and Lys66 is to our knowledge present only in HLA-A2. A majority of alleles from the HLA-A group have an arginine at position 65, besides HLA-A*23 and HLA-A*24 which both have a glycine at this position. However, these two latter antigens still have a lysine at position 66 (Lys66) which, according to the crystal structures, can bind to a presumptive phosphate (Figure 3). At this stage, we can only speculate that additional phosphopeptides can be presented similarly by a larger array of MHC-I alleles, and that this anchoring pattern is not only restricted to HLA-A2.

HLA-B antigens do not have an arginine at position 65. Instead, most HLA-B alleles have an arginine at position 62 (Arg62) except for HLA-B*57 and HLA-B*58 which both have a glycine (Gly62). 256 unique phosphopeptides associated with HLA-B*40, -B*27, -B*39 or -B*07 were recently identified and analyzed (Alpízar et al., 2017), demonstrating again that although the eluted peptides have different anchors, which is expected since they are restricted to different HLA-B alleles, a phosphate modification was found in most cases at peptide position 4. Again, many phosphopeptides also had a basic residue at p1. The crystal structure of HLA-B*40:02 in complex with the peptide REFsKEPEL, derived from the inner centromere protein provided first insights into binding features of phosphopeptides to HLA-B molecules (Alpízar et al., 2017). The high prevalence of phosphorylation at peptide p4 is dictated by the conserved Arg62, a structural feature that is shared by most HLA-B alleles. In contrast to HLA-A2, the preference for basic residues at p1 is more difficult to explain in HLA-B alleles, since the phosphate moiety does not make any bond with the first peptide residue.

### 4.3 Citrullination

Citrullination is a post-translational modification in which an arginine residue is converted to citrulline (R-NH-C(NH_2_) = NH^+^→ R-NH-C(NH_2_) = O) by peptidyl-arginine deiminase enzymes that remove the positive charge of arginines. Citrullination is intensively studied within the frame of a large array of autoimmune diseases (Zavala-Cerna et al., 2014). Indeed, it is well-established that citrullination of self-antigens is strongly associated with RA, as demonstrated initially by the identification of autoantibodies directed to citrullinated proteins in patient’s sera (James et al., 2010). Analysis of C1R cell lines expressing HLA-A*01:01, HLA-A*02:01 or HLA-A*24:02 demonstrated that about 3% of PTM peptides presented by these common MHC-I allotypes were citrullinated (Mei et al., 2020). In contrast to glycosylation and phosphorylation, citrullination does not change the shape of the PTM peptide but instead modifies significantly the electrostatic potential of the side chain, affecting interaction properties of the modified epitope.

**FIGURE 4:**
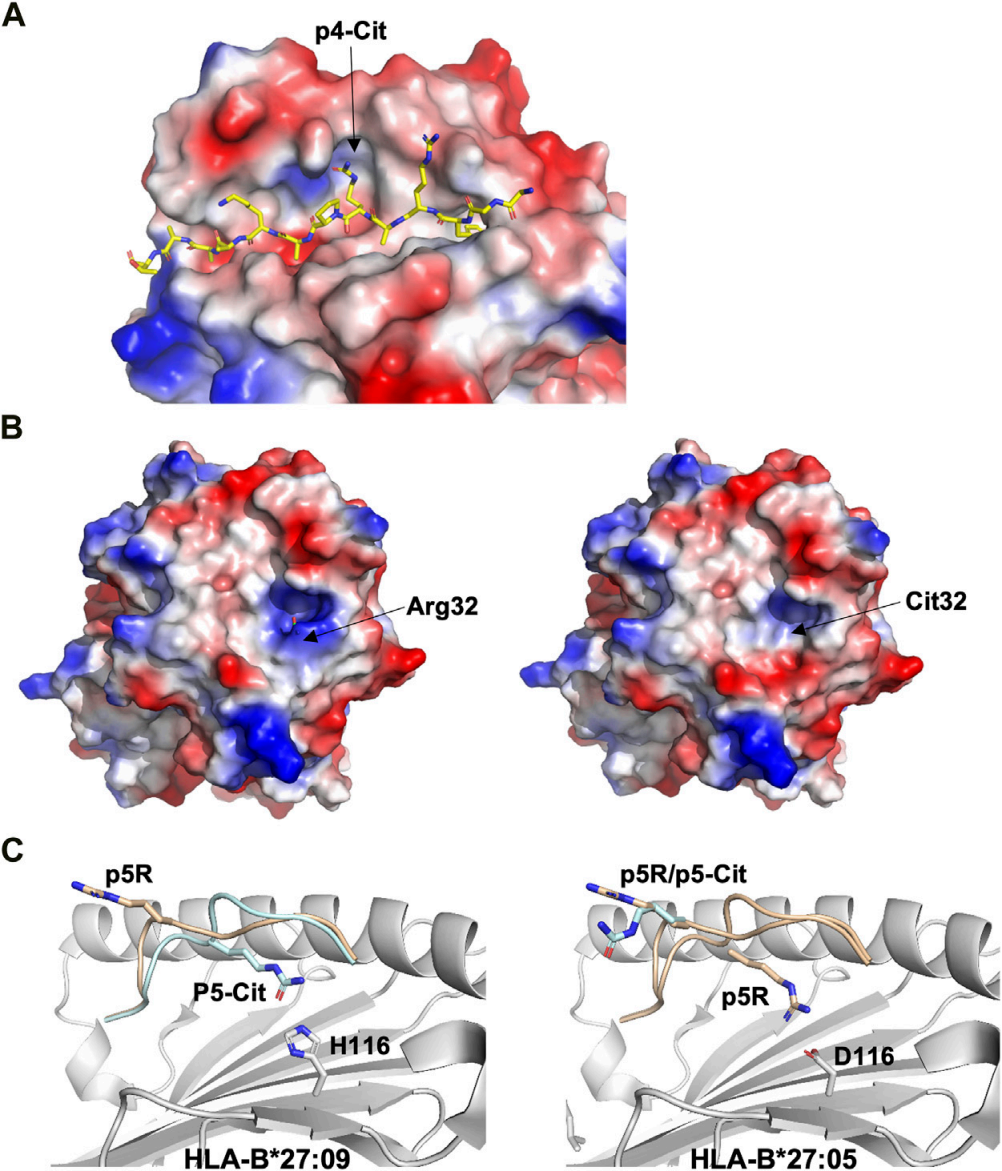
Citrullination may allow binding of neoantigens to MHC, modify surface electrostatics and alter the conformations of presented peptides **(A)**. The side chain of a citrullinated arginine residue can bind to the slightly positively charged pocket 4 in HLA-DRB1*04:01 while the side chain of an arginine residue will be repelled, as exemplified here by the crystal structure of the citrullinated fibrinogen peptide (6BIL.pdb) (Ting et al., 2018). The peptide, colored in yellow, binds to the cleft of HLA-DRB1*04:01 with the N- and C-termini extending to the left and right of the peptide binding cleft, respectively. The side chain of the citrullinated residue at p4 is indicated, binding to the slightly positively charged pocket 4 in HLA-DRB1*04:01. The surface of HLA-DRB1*04:01 is colored according to surface electrostatics, with negatively and positively charged regions in red and blue, respectively **(B)**. Citrullination of peptides may result in modification of the pMHC surface electrostatic potential, as exemplified by the surfaces of HLA-DRB1*04:01 in complex with the wild-type enolase-derived epitope eno_26-40_ (5LAX.pdb, left) or with the citrullinated PTM variant (5JLZ.pdb, right) (Gerstner et al., 2016). Here, citrullination reduces significantly the size of the positively charged region, possibly leading to the selection of alternative TCRs. Both structures are presented from the TCR view **(C)**. The conformation of the self-peptide VIRP_400-408_ depends on the MHC-I allele it binds to. Wild-type VIRP_400-408_ binds to HLA-B*27:09 with the side chain of the arginine residue p5R protruding towards the solvent and a presumptive TCR (1OGT.pdb, colored in light orange, left panel). Citrullination of p5R in VIRP_400-408_ modifies significantly the conformation of the PTM peptide compared to wild-type VIRP_400-408_. The side chain of p5R in the citrullinated VIRP_400-408_ dives instead within the cleft of B*27:09 forming hydrogen bonds with the side chain of H116 (3B3I.pdb, colored in blue, left panel). VIRP_400-408_ takes two different conformations when binding to HLA-B*27:05, one similar to the one described for the HLA-B*27:09/VIRP_400-408_ complex and one diametrically different in which the peptide flips and the side chain of p5R forms hydrogen bonds with the side chain of the HLA-B*27:05 aspartate residue D116 (1OGT.pdb, colored in light orange, right panel). Importantly, citrullination of p5R forces the formation of only one peptide conformation in which the side chain of the citrulline residue protrudes towards the solvent (3B6S.pdb, colored in light blue, right panel) (Beltrami et al., 2008).

Three major anchor residues, localized at p1, p4 and p9, play a key role in peptide binding to HLA-DRB1 molecules. While the first peptide residue must be both large and hydrophobic in order to bind to pocket 1 into HLA-DRB1, the other two pockets are more polymorphic among different alleles. For example, while pocket 4 in DRB1*0401 or DRB1*0404 is positively charged and can therefore accommodate acidic or polar residues, DRB1*0402 comprises two acidic residues that line pocket 4, resulting in a preference for non-basic residues. These differences in pocket 4 are considered to be one of the main causes for the association of MHC-II alleles such as HLA-DRB1*0401, DRB1*0101 or DRB1*0404 with RA (Scally et al., 2013). Structural studies have demonstrated that citrullination of an arginine residue at p4 can result in the creation of a neo-self-antigen since this PTM makes it possible for the modified epitope to bind to HLA-DRB1*0401 or HLA-DRB1*0101, while the wild-type peptide cannot (Figure 4A) (Ting et al., 2018).

Citrullination of TCR-interacting peptide residues may also result in the formation of neoepitopes that affect TCR recognition. The crystal structures of the DRB1*0401-restricted α-enolase-derived peptide eno_26-40_ (TSKGLF**R**AAVPSGAS) with the arginine residue Arg32 located at position 2 within the peptide core were determined both for the wild-type and citrullinated peptide variants (Gerstner et al., 2016). The two pMHC complexes are almost identical, with few specific differences in the electrostatic potential of their surfaces (Figure 4B). These localized electrostatic differences allow for the selection of potentially autoreactive CD4^+^ T cells. Furthermore, citrullination of residues positioned on the end-edges of the peptide binding cleft can also cause different functional results. For example, the crystal structure of DRB1*0401/eno_326-340_ demonstrated that the citrullinated residue is located at peptide position -1 (Pieper et al., 2018). Here again, although the structures of wild-type and citrullinated pMHC are almost identical, the frequency of CD4^+^ T cells recognizing DRB1*0401/cit-eno_326-340_ was significantly elevated in synovial fluids compared to peripheral blood (Pieper et al., 2018). Thus, citrullination of peptide residues localized either centrally or on the edges of MHC-II clefts may both promote higher binding affinity and create neoantigens that induce T cell autoreactivity.

Ankylosing spondylitis (AS) is strongly associated to HLA-B*2705, and it has been demonstrated that peptide citrullination can significantly alter the binding mode of the modified epitope (Beltrami et al., 2008). It is also well-established that while HLA-B*2705 is associated with AS, HLA-B*2709 is not, although these MHC-I differ by only one aa residue at position 116. Residues Asp116 in HLA-B*2705 and His116 in HLA-B*2709 sit in the middle of the peptide-binding cleft. It has been demonstrated that the D116H mutation affects the conformation of the self-peptide VIPR_400-408_ (RRKWRRWHL) derived from the vasoactive intestinal peptide type I receptor (Beltrami et al., 2008). VIRP_400-408_ binds canonically to HLA-B*2709, resulting in the extension of the side chain of the arginine residue at p5 towards a presumptive TCR (Figure 4C). In contrast, the same residue can take two different conformations whence in complex with HLA-B*2705. While the first conformation in HLA-B*2705 is very similar to that found in HLA-B*2709, the second one is entirely different with the formation of salt bridges between p5R and Asp116 (Figure 4C) (Hülsmeyer et al., 2004). Citrullinated p5R is an anchor residue in HLA-B*2709 while it is solvent exposed when in complex with HLA-B*2705 (Figure 4C) (Beltrami et al., 2008).

MHC-restricted self-epitopes that trigger TCR autoreactivity display also often low binding affinity compared to viral and/or bacteria peptides. Indeed, 75% of known type I diabetes-associated antigens bind their cognate HLA with an average affinity of 8,250 nM or higher, which is almost 20 times lower compared to pathogen-derived peptides (average affinity of 500 nM for 85% of epitopes) (Sidney et al., 2018). Common modifications in *e.g.* insulin-B-derived peptides, such as citrullination or chlorination improved significantly the binding capacity of these foreign epitopes to MHC molecules which is a plausible non-excluding explanation for T-cell activation in type I diabetes, leading to the killing of beta cells (Sidney et al., 2018).

Besides the established importance of citrullination in autoimmune diseases, this PTM has also attracted great attention within the frame of tumor immunology, since cancer cells can overexpress protein arginine deiminase (PAD) enzymes upregulating autophagy (Mondal and Thompson, 2021), which results in presentation of MHC-II-restricted citrullinated peptides that could be used as novel targets for immune therapy (Cook et al., 2018; Brentville et al., 2020). Potent CD4 responses have been described against citrullinated pMHC complexes in both human studies and in HLA transgenic mouse models. The anti-tumour effect relied upon direct recognition of tumours by specific CD4^+^ T cells, suggesting that citrullinated peptides are attractive targets for cancer vaccines (Cook et al., 2018).

### 4.4 Nitration

Addition of an NO_2_^−^ group to tyrosines or tryptophans occurs spontaneously in cells under oxidative stress (Petersen et al., 2009a). Activation of macrophages and dendritic cells includes the production of nitric oxide and other reactive oxygen species. These compounds are also formed *in vivo* at sites of inflammation and in a wide array of diseases, including Alzheimer, atherosclerosis, stroke and cancer. Peroxynitrous acid (ONOOH), a potent oxidizing and nitrating agent, is formed from superoxide and nitric oxide radicals. Exposure of proteins to these compounds can lead to tyrosine and tryptophan nitration (Nuriel et al., 2011). CD4^+^ T cell subsets can specifically recognize nitration modifications in both tyrosine and tryptophan residues in I-A^k^-restricted epitopes derived from the hen egg-white lysozyme protein (Herzog et al., 2005). Elevated levels of nitrated residues are routinely used as indicators for inflammation and tumor progression (Ene et al., 2020). Nitrated proteins are present in inflammatory sites in autoimmune diseasess, various cancers and infectious diseases (Madhurantakam et al., 2012).

The consequences of nitrotyrosination on binding affinity of peptides to MHC-I and their effects on TCR recognition have been studied using the LCMV infection model system. Nitrotyrosination of the immunodominant LCMV epitopes gp33 (KAV**Y**NFATC/M) and gp34 (AV**Y**NFATC/M) alters profoundly T cell recognition in the context of H-2D^b^ and H-2K^b^ (Achour et al., 2002; Velloso et al., 2004; Hardy et al., 2008). Comparative analysis of the crystal structures of H-2K^b^/gp34 and H-2K^b^/NY-gp34 demonstrated that nitrotyrosination of p3Y in gp34 abrogates a hydrogen bond formed with the H-2K^b^ TCR-interacting glutamate residue E152 changing its conformation and modifying the surface of the nitrotyrosinated pMHC (Madhurantakam et al., 2012). Furthermore, nitrotyrosination of gp34 resulted in structural over-packing, reducing the stability of H-2K^b^/NY-gp34 compared to H-2K^b^/gp34. Nitrotyrosination of the main TCR-interacting residue p4Y in gp33 abrogates recognition of H-2D^b^/gp33-NY complexes by H-2D^b^/gp33-specific T cells. All these functional effects could be favorable for the capacity of LCMV to escape from CD8^+^ T cell recognition.

### 4.5 Lipidation

**FIGURE 5:**
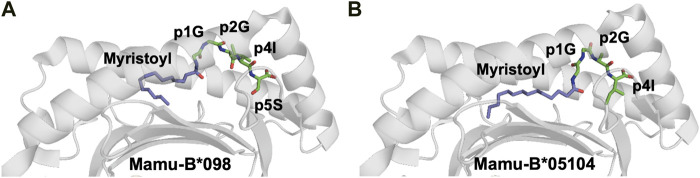
The myristoyl moiety in myristoylated peptides plays an essential role in both MHC-I binding and adequate presentation of the epitope **(A)**. The crystal structure of the rhesus macaque MHC-I molecule Mamu-B*098 in complex with the myristoylated 5-mer lipopeptide derived from SIV Nef protein (4ZFZ.pdb), reveals the dual role of the PTM moiety in both adequate binding to the MHC-I and peptide presentation (Morita and Sugita 2016). **(B)**. The crystal structure of the rhesus macaque MHC-I molecule Mamu-B*05,104 in complex with a N-myristoylated 4-mer lipopeptide derived from the SIV nef protein (6IWG.pdb) shows how the myristoyl moiety occupies the cleft differently (Yamamoto et al., 2019)

A novel MHC class I-restricted lipopeptide antigen was recently identified (Yamamoto et al., 2019). Cellular proteins comprising an N-terminal Gly-x-x-x-Ser/Thr motif (where x is any amino acid) can undergo N-myristoylation, in which N-myristoyltransferase catalyzes the conjugation of a 14-carbon fatty acid (myristic acid) to the N-terminal glycine residue, using myristoyl-CoA as substrate. N-myristoylation can occur in both host and viral proteins in virus-infected cells. The rhesus macaque CD8^+^ cytotoxic T cell line 2N5.1 specifically recognizes simian smmunodeficiency virus (SIV) Nef protein-derived N-myristoylated 5-mer peptides (C14-Gly-Gly-Ala-Ile-Ser [C14nef5]) that bind to the macaque MHC-I allele Mamu-B*098. The crystal structure of the Mamu-B*098/lipopeptide complex revealed that the myristic group and the conserved C-terminal residue of the lipopeptide serve as anchors, whereas a short central stretch of three aa residues can interact with TCRs (Figure 5) (Morita and Sugita, 2016; Yamamoto et al., 2019).

### 4.6 Peptide splicing and the immunopeptidome

**FIGURE 6:**
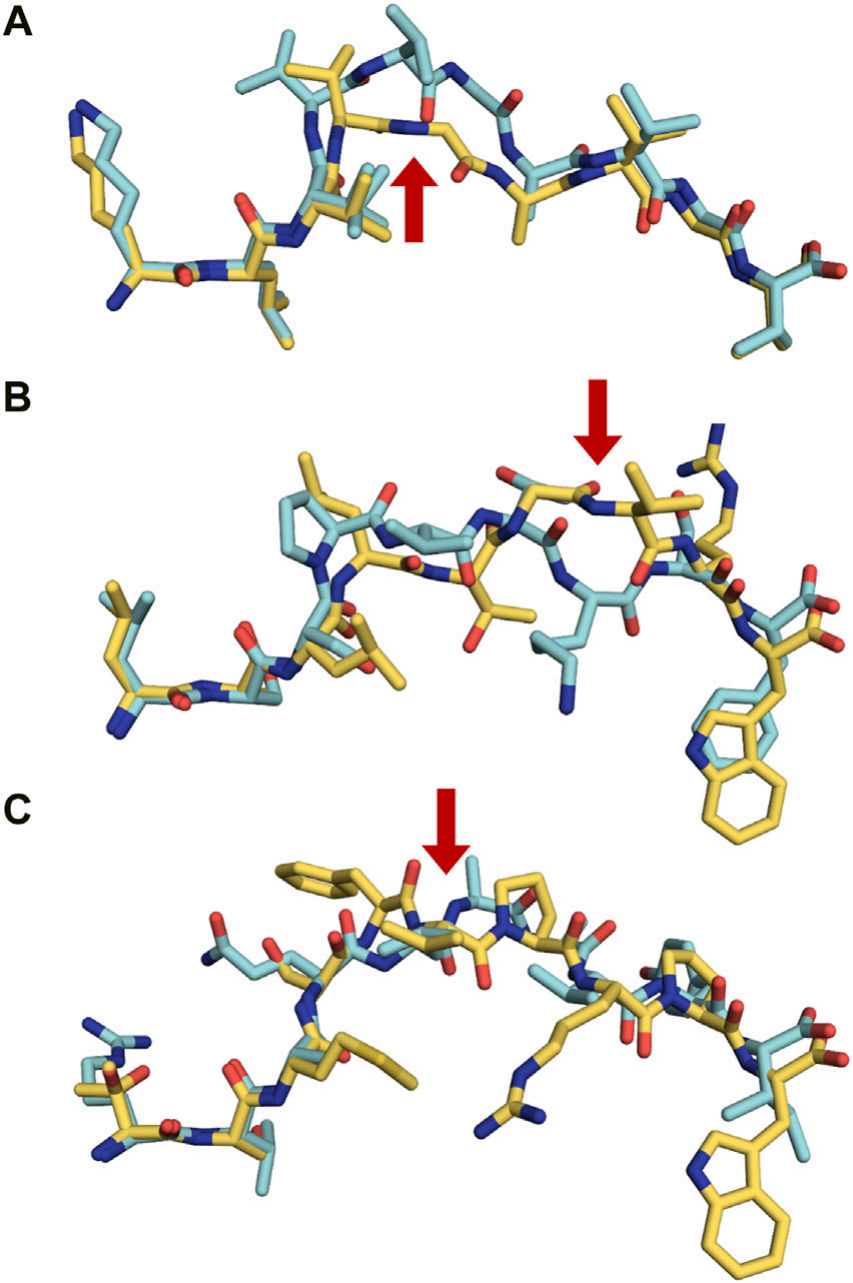
Proteasomally spliced peptides bind in a canonical mode to MHC molecules **(A)** Comparison of the conformations of the original KRAS_5-14_ peptide (6O53. pdb, cyan) and its cis-spliced variant KRAS_5-6/8–14_ (6O4Y.pdb, yellow) both in complex with HLA-A2*01 demonstrates that the splicing results in the formation of a neoepitope through the removal of a central conformational bulge following splicing (Mishto et al., 2019). The red arrow shows the junction between spliced fragments. **(B)** Although sequentially unrelated, a comparison of the linear nonameric self-peptide 94-LSSPVTKSF-102 derived from immunoglobulin kappa (2RFX.pdb, cyan) (Chessman et al., 2008) and the nonameric cis-peptide LALLTGVRW (6D2T.pdb, yellow) reveals that i) they make use of similar anchor residues (p2A/p2S and pF9/pW9) in order to bind to HLA-B*57:01 (Faridi et al., 2018; Mishto et al., 2019), and ii) that the junction between spliced fragments is protruding towards the solvent, readily available for interactions with TCRs (red arrow) **(C)**. A similar comparison is presented here with two unrelated decameric peptides. The junction between spliced fragments in the trans-spliced epitope TSMSFVPRPW (6D29. pdb, yellow) (Faridi et al., 2018) is also available for interactions with TCRs. The HLA-B*57:01-restricted decameric peptide from small nuclear protein SmD3 54-RVAQLEQVYI-63 (3VRI.pdb) is presented as a reference for a conventional epitope (Illing et al., 2018). The red arrow shows the junction between spliced fragments.

MHC-presented peptides have been considered for many years as only linear fragments derived from full length proteins. However, it was established 15 years ago that proteasomes also produce spliced peptides which are composed from two or several fragments of the same protein (cis-splicing) (Hanada et al., 2004) or derived from different proteins (trans-splicing) (Faridi et al., 2018). Trans- and cis-spliced peptides occur with comparable abundance in immunopeptidomes, and these proteasome-spliced epitopes are able to trigger *in vivo* CD8^+^ T-cell-mediated responses (Vigneron et al., 2004; Warren et al., 2006). It is obviously much more difficult to identify and assess the overall quantity of this family of peptides using MS analysis. An ongoing controversy raised by contrasting results from different research groups about the frequency (with estimates that vary from less than 1% to 25–30%), the abundance and even whether spliced epitopes (splicetopes) do represent only a very rare curiosity is discussed in a recent review (Kloetzel, 2022). Usage of theoretical libraries of cis-spliced peptides revealed that they could constitute about a third of the entire antigenic peptide pool in human EBV-transformed cell lines and primary fibroblasts (Liepe et al., 2016). Of the about 50,000 peptides identified in 17 monoallelic cell lines, 72% were conventionally linear, while 25% were either cis- or trans-spliced variants, and 3% remained uncharacterized. Interestingly, the fraction of spliced-peptides seems to be allele-specific corresponding for example to 44.7% of all identified peptides for HLA-B*15:02 while only 12.6% for HLA-A*24:02. The average length (8–12 residues) and binding motifs of spliced peptides are similar to linear epitopes for most alleles, although it should be noted that servers such as NetMHC predict 77% of linear peptides and only 47% of the spliced variants, a problem that therefore required the development of a novel prediction algorithm (Faridi et al., 2018). Several crystal structures of MHC-restricted spliced peptides have been determined, demonstrating a conventional binding with canonical anchor residues (Figure 6) (Mishto et al., 2019). Furthermore, structural analysis of trans-spliced peptides also revealed that the junction between spliced fragments is often highly solvent exposed and is likely to participate in interactions with TCRs (Figures 6B,C) (Faridi et al., 2018). Altogether, these results demonstrate a much higher diversity of the immunopeptidome than previously expected, which we believe should be taken into consideration within the frame of immunotherapy and vaccine design.

## 5 Post-translational modifications as neo-antigens

Immunotherapy is a relatively novel tool for treating cancers, in which the immune system is triggered to recognize and control cancerous tumors with high specificity. The development of checkpoint blockade therapy, including anti-PD-1 and anti-CTLA-4 antibodies has provided a new weapon against cancer, which enhances significantly anti-tumor immune responses. Adoptive T cell therapies can be used, alone or in combination with checkpoint inhibitors, to selectively enhance T cell responses towards MHC-restricted tumor-associated antigens (TAA) and/or tumor-specific antigens (neoantigens) presented on tumor cells (Cable et al., 2021). While TAAs are derived from proteins that are overexpressed in tumors compared to healthy tissues, neoantigens are most often products of somatic mutations or derived from oncoviruses, and are not found in healthy cells. In our opinion, although the identification of neoantigens is still challenging, the combination of analyses of genome and transcriptome using next generation sequencing technology (Wu et al., 2018), combined with direct molecular evidence provided by mass-spectrometry of the true presence of MHC-restricted neoantigens has opened for formidable possibilities to unambiguously identify cancer- and other disease-associated neoantigens (Zhang X. et al., 2019). Furthermore, it should also be noted that an alternative antigen repertoire of T cell epitopes associated with impaired peptide processing, called TEIPP antigens, has been recently discovered. These MHC-restricted non-mutated peptides, derived from housekeeping proteins, are not presented in homeostasis (Wolpert et al., 1997; Chambers et al., 2007; Kiessling 2016; Marijt et al., 2019a; Marijt et al., 2019b). TEIPPs are only presented on the surface of cells with significantly reduced pMHC levels, following mutation/deletion of proteins essential for antigen processing. The crystal structure of the first identified TEIPP peptide, Trh4 (van Hall et al., 2006), revealed that it takes a conventional conformation within H-2D^b^, although its does not comprise the prototypic p5N/p9M motif (Hafstrand et al., 2016; Hafstrand et al., 2018). It remains to our knowledge unclear whether the TEIPP antigen family comprises epitopes with post-translational modifications.

The database TSNAdb (http://biopharm.zju.edu.cn/tsnadb) contains already about 1.3 million tumour associated antigens and neoantigens. Importantly, a large amount of the 14,000 listed PTM epitopes could be cancer-associated. Indeed, 1,742 MHC-I- and 1,709 MHC-II-restricted PTM antigens were detected in at least one cancer sample but not in any of the 707 analysed non-cancerous samples, except for testis. 23 MHC-I and 15 MHC-II PTM epitopes were identified in more than seven different cancer samples, almost all derived from overexpressed proteins in tumor samples (Yi et al., 2021). Interestingly, the PTM frequency appears to be cancer dependent, with *e.g.* glutathionylation representing 66% of all PTM in lymphoma cells. It should however be noted that acetylation is the most frequent PTM in all other types of cancer.

Transformation of a cell from healthy to tumor is accommodated by changes in metabolism (Duraj et al., 2021). In particular, malignant cells display most often altered glucose metabolism since ATP is produced through glycolysis instead of oxidative phosphorylation (Ghanem et al., 2021). Glycan synthesis is a highly regulated multistep process that depends upon the availability of several glycosylation enzymes, as well as substrates and sugar donors. Changes in carbohydrate metabolism and enzyme expression levels may alter glycan synthesis in cancer cells, resulting in truncated O-glycans, abnormal branched N-glycans, diverse fucosylation and increased sialic acid on proteins (Mereiter et al., 2019). Thus, these modifications in glycosylation process can result in the expression of PTM epitopes, as already demonstrated in several studies. 80 unique MHC-II glycosylated peptides derived from 28 different proteins were found in three melanoma cell lines and a matched EBV-transformed cell line (Malaker et al., 2017a). Interestingly, 62 glycopeptides formed nested sets, with the same nine residues binding core and different numbers of flanking residues. Only eight glycopeptides were detected in non-melanoma EBV-transformed cells. The glycosylation pattern was extremely diverse and complex, with 17 different identified glycoforms, and 82% were N-linked to asparagines. Different mannose-rich branched glycans containing up to six different sugars, and sometimes consisting of 12 sugars were found. Furthermore, a vast majority of the glycosylations occured on flanking peptide residues, stretching most probably out from the peptide binding clefts. Three-dimensional molecular models indicated that the glycan moieties could still contact and thus modulate interactions with TCRs (Malaker et al., 2017a).

Although cancers such as leukemia and renal carcinoma display very low mutational loads, these cancers respond usually well to immunotherapy (Lawrence et al., 2013). It has been suggested that PTM rather than somatic mutations are mainly responsible for the production of MHC-restricted neoantigens in leukemia. To address this hypothesis, MS analysis was specifically performed on *O-*glycosylated peptides (Malaker et al., 2017b). 36 HLA-B*07:02-restricted *O-*GlcNAcetylated peptides were identified in leukemia samples, of which 33 were not present in healthy tissue cells. Most often, PTM occurred on the fourth or fifth residue, similarly to previous results in mouse models (Figure 2). This indicates a functional importance for these modifications since the side chains of p4 and p5 usually interact with TCR CDR3. Indeed, an *O*-GlcNAc-specific T-cell line killed efficiently autologous cells pulsed with the PTM peptide, but not the unmodified epitope. Importantly, many identified glycosylated peptides were immunogenic and recognized by cytotoxic and memory CD8^+^ T cells. Furthermore, T cells from healthy donors specifically targeted and killed only cells that displayed PTM peptides (Malaker et al., 2017b).

An interesting example of a MHC-I-restricted neoantigen derived from glycosylated p53 has also been described (Nguyen et al., 2021). The authors tried to explain the observed differences between cancer patients and healthy donors in their capacities to induce CD8^+^ T cell responses towards a p53-derived HLA-A*24:02-restricted epitope. Indeed, efficient and specific cytotoxic T cell responses were obtained upon incubation of peripheral blood mononuclear cells (PBMC) from hepatocellular carcinoma or pancreatic adenocarcinoma HLA-A*24:02^+^ patients with the synthetic p53-derived peptide p161 (AIYKQSQHM_161-169_). The same peptide did not elicit any immune response in PBMC cells from healthy HLA-A*24:02^+^ donors (Mizukoshi et al., 2011), leading the authors to suggest a mechanism in which the potentially naturally occurring glycosylation of the serine residue at peptide position 6 could be omitted in cancer cells (Bennun et al., 2013; Peixoto et al., 2019), resulting in the formation of a unglycosylated neoepitope. Since glycosylation occurs at the sixth peptide position in the HLA-A*24:02-restricted peptide, the glycan moiety should protrude towards the solvent and be fully accessible for interactions with adequate TCRs (Nguyen et al., 2021).

Phosphorylation is another well-studied main source of MHC-restricted PTM peptides that are of interest for immunotherapy. It is well established that phosphorylation is an obligatory step in immunological pathways, and several cancers exhibit oncogenic activation of kinase signaling (Aoki and Fujishita 2017; Du and Lovly, 2018; Rane and Minden, 2019) or dysregulation of this pathway (Newton, 2018). Different types of cancer display different amounts of phosphopeptides, usually derived from different proteins. However, to our knowledge, not a single MHC-I phosphopeptide has yet been identified in colon carcinoma, while only one was identified in breast cancer. A total of 13, 20 and 36 MHC-bound phosphopeptides were recently identified in glioblastoma, lymphoma and melanoma (Yi et al., 2021). However, the differences observed among tumors could be due to the relative haplotypes of the studied cells. The amount of HLA-B-restricted phosphopeptides in melanoma cells are 4–5 times larger compared to HLA-A antigens (Zarling et al., 2000).

## 6 Use of unnatural amino acids as a scientific tool

Chemically modified unnatural aa have been previously used to address the structural bases underlying modulation of TCR recognition. Pioneering experiments were performed using the HLA-A2-restricted human T cell lymphotrophic virus-1 (HTLV-1)-derived Tax peptide (LLFGY**P**VYV). The P6A-Tax variant in which the proline at p6 is mutated to alanine is a TCR antagonist. Comparison of the crystal structures of the TCR A6 in complex with either HLA-A2/P6A-Tax or HLA-A2/Tax revealed that these ternary complexes were nearly identical besides a slight packing defect above P6A-Tax, with a cavity partially filled by a water molecule (Ding et al., 1999). A follow-up study made use of the unnatural amino acid variants N-methyl-glycine, N-ethyl-glycine and N-propyl-alanine in order to replace progressively the gap formed by the P6A mutation, filling stepwise the pocket above the peptide. The results of the study demonstrated an elegant progressive raise in affinity of the TCR to the different pMHC complexes, which corroborated with receding antagonistic effects on the same T cell clone (Baker et al., 2000).

**FIGURE 7:**
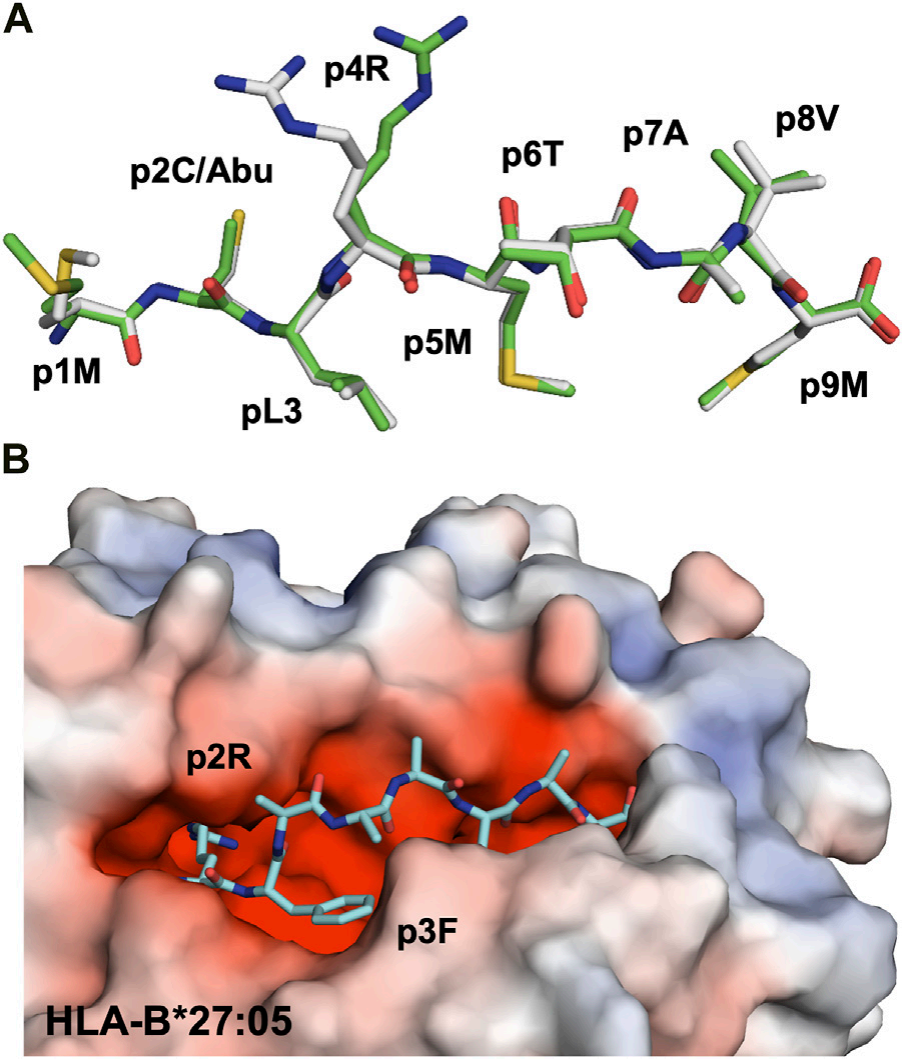
Unnatural amino acids can be easily used to assess the importance of peptide residues for pMHC stability and TCR recognition **(A)** The unnatural aa α-aminobutyrate was used to replace a cysteine residue, abolishing sulfur–π interactions with this peptide residue and the H-2D^b^ residue Y45 in H-2D^b^/Trh4, demonstrating the key role of this type of interaction for the efficient binding of this TEIPP peptide to H-2D^b^. Comparison of the crystal structures of H-2D^b^ in complex with Trh4 (5E8N.pdb) or Thr4-p2ABU (5E8O.pdb) demonstrated that the conformation of the backbone of the altered peptide (green) is identical to wild-type Trh4 (grey) despite significant different overall pMHC stability (Hafstrand et al., 2016) **(B)** Replacement of two anchor residues in the peptide bound to HLA-B*27:05 (Hülsmeyer et al., 2004) with unnatural amino acids (1JGE.pdb) resulted in the destabilization of MHC/peptide complex and decreased immunogenicity (Jones et al., 2006).

More recently, unnatural aa were used to identify the anchor motif in the first identified TEIPP epitope Trh4, which is rich in residues comprising sulfur atoms (**MC**LR**M**TAV**M**) (Hafstrand et al., 2016). Although lacking the prototypic asparagine anchor residue p5N which is a key part of the anchor motif for H-2D^b^-restricted peptides, Trh4 binds with high affinity to this MHC-I. The crystal structure of H-2D^b^/Trh4 revealed that the TEIPP epitope takes a noncanonical binding pattern with extensive SH-π interactions that contribute to the overall complex stability. The noncanonical methionine p5M acts as a main anchor, altering only the conformation of two heavy chain residues Y156 and H155, thereby forming a unique pMHC conformer that was hypothesized as essential for recognition by TEIPP-specific T cells. Indeed, mutation of p5M in Trh4 to a conventional asparagine abolished recognition by the H-2D^b^/Trh4-specific T cell clone LnB5. The key role of sulfur atoms in methionines and cysteines was elucidated by replacing them with norleucine and α-aminobutyrate, respectively (Hafstrand et al., 2016). Although replacement of a single sulfur atom in p2C with a CH_2_-group found in α-aminobutyrate did not alter peptide conformation, it reduced significantly the stability of the pMHC, demonstrating the key role of SH-π interactions (Figure 7A) (Hafstrand et al., 2016).

The association of HLA-B27 with the inflammatory rheumatic disorder ankylosing spondylitis (AS) was established for more than 50 years ago (Brewerton et al., 1973). While HLA-B27:02, HLA-B27:05 and HLA-B27:04 predispose to AS, the alleles HLA-B27:06 and HLA-B27:09 are not associated to the disease (Khan 2013). Interestingly, the majority of HLA-B27 alleles associated with AS display a reduced overall stability compared to other non-associated HLA-B27 or to other MHC-I alleles (Chen et al., 2017). The AS-associated allele HLA-B*2705, binds preferentially peptides containing an arginine at p2. In order to increase the affinity of peptides for HLA-B*2705, two altered peptide ligands (APL) were synthesized in a recent study in which arginine p2R was substituted to the α-methylated β,γ-unsaturated arginine analogue 2-(S)-amino-5-guanidino-2-methyl-pent-3-enoic acid (Jones et al., 2006). The modified peptides displayed lower affinity to HLA-B*2705 compared to native epitopes, most probably due to improper electrostatic interactions, and elicited weaker cytotoxic responses from cognate epitope-specific CD8^+^ T-cells (Figure 7B). Another study focused instead on the molecular details of epitope anchoring to HLA-B27 by assessing the role of the aromatic residue at p3. Three unnatural aromatic aa were introduced in the *Chlamydia trachomatis* GroEL_117–125_ peptide (KRGIDKAAK) in attempts to create analogues with higher binding affinity and increased pMHC stabilization capacity (Krebs et al., 1998). Three-dimensional molecular models suggested that homophenyl-alanine and naphthylalanine at p3 may fit optimally to the hydrophobic D pocket in HLA-B27. Thermal denaturation experiments demonstrated that the overall stability of HLA-B27 in complex with synthetic APLs was higher compared to the wild-type peptide.

An innovative approach to facilitate high-throughput structural determination has been previously suggested by Schumacher and colleagues (Celie et al., 2009). The authors designed UV-sensitive epitopes, in which peptide bonds could be cleaved following short exposure to UV light. As a result, an MHC-bound reference UV-sensitive peptide is cleaved into low affinity fragments that can be exchanged *in-crystallo* against another full-length epitope. Crystal structures of the same pMHC determined using a conventional X-ray crystallography approach and the one suggested by Celie *et al* demonstrated clear similarities, thus proving the quality of this innovative approach. It should be noted that the same technology is also commonly used for the production of MHC-I tetramers and in a large array of functional identification assays (Lampen et al., 2018).

Wilms tumor protein (WT1) is a zinc-finger transcription factor that is selectively overexpressed in leukemia and other types of cancers, and it has been suggested that several WT1-derived peptides could be used as a vaccine (Jiang et al., 2021). Thirteen variants of WT1 peptides were synthesized with chemically modified aa via fluorination and photo-reactive group additions at both MHC and T cell receptor binding positions. Here again, several non-natural peptide analogs stabilized pMHC-I complexes better than native epitopes. Moreover, these APLs elicited specific T-cell responses and in some cases high cytotoxicity towards leukemia cells (Gómez-Nuñez et al., 2008).

## 7 Use of chemically modified peptides as a medical tool

Peptide vaccination can be used in cancer treatments as well as against specific infectious diseases (Richman et al., 1979; van Hall and van der Burg 2012). A major factor that may limit the efficiency of peptide vaccines *in vivo* is their often high propensity for rapid degradation in serum as well as other biological fluids (Powell et al., 1992). Incorporation of peptide bond surrogates in MHC epitopes could be a useful approach to design less degradable APLs that bind with high affinity and enhance pMHC stability (Guichard et al., 1996). Altogether, we believe that depending on their agonist or antagonist effects towards T cells, such non-natural MHC ligands could most likely be used efficiently in a large ensemble of applications both for enhancing responses or in other cases eliciting antagonistic responses that could hamper unwanted T cell responses. Development of peptide-based vaccines could thus represent a possibility to further enhance responses towards different kinds of tumors, or could conversely be used as a potential therapeutic agent in treatment of allergies and autoimmune diseases.

**FIGURE 8:**
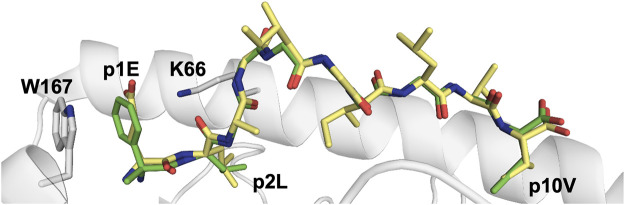
Replacement of three residues in the HLA-A*02:01-restricted melanoma epitope Mart-1 with unnatural amino acids did not alter the conformation while enhancing binding affinity.Comparison of the crystal structure of the wild-type Mart-1 peptide ELAGIGILTV (1JF1. pdb, grey) with the APL in which p1E, p2A and p10V were modified to phenylglycine, norvaline and PRG, respectively (4WJ5. pdb, green) (Hoppes et al., 2014). Two HLA-A*02:01 residues that interact with the modified residues in the APL are also indicated.

A non-natural, peptidase-resistant antigenic analogue of the human tumor antigen MAGE-1. A1 (EADPTGHSY) has been previously designed (Ayyoub et al., 1999). Since the residues located at p1 and in the central p4–p8 region are recognized by anti-MAGE-1. A1 CTL clones, the remaining residues were modified in order to design more stable, while biologically still active non-natural MAGE-1. A1 analogues. The APL E (Aib)DPTGH(NMe-Ser8)Y was highly peptidase-resistant, bound efficiently to HLA-A*0101 and activated MAGE-1. A1-specific anti-melanoma CTLs (Hoppes et al., 2014). Several additional epitopes were screened with more than 90 protease-resistant synthetic aa, which resulted in a total of about 3,000 chemically enhanced APLs with good binding affinity to HLA-A*0201. The crystal structure of one such modified MAGE-1. A1 analogue is presented in Figure 8. Three modifications in which residue p1E was substituted to phenylglycine, p2A to norvaline and p10V to a proteinogenic amino acid (PRG), resulted in ID_50_ values about 100 times lower compared to the native epitope and 10 times lower compared to the conventionally modified APL A2L in which alanine at p2 was mutated to a leucine. The enhanced affinity is explained by 1) enhanced interactions between p1 and specific heavy chain residues, forming π−π interactions with the aromatic ring of residue W167, and cation-π interactions with K66, and 2) a more snuggly fit within hydrophobic pockets of HLA-A*0201 by PRG residues (Hoppes et al., 2014).

A similar approach was also taken on HLA-A*0201-restricted influenza-derived epitopes. Here again, a large amount of chemically enhanced APLs were designed for GILGFVFTL, FMYSDFHFI and NMLSTVLGV (Rosendahl Huber et al., 2016). Although these three viral epitopes are immunodominant and already displayed high binding affinity to HLA-A*0201, some of the designed unnatural APL variants exhibited significantly enhanced binding affinity compared to their wild-type counterparts. For example, insertion of the non-proteogenic aa d-α-methyl-phenylglycine at p1 in GILGFVFTL improved binding affinity to HLA-A*0201 by 14%. Moreover, vaccination of HLA-A*0201 transgenic mice with selected APL variants resulted in increased IFN-γ responses by splenocytes.

GalNAc-glycopeptides derived from the polymorphic epithelial mucin MUC1 are an important class of TAAs. Attempts to modulate peptide/carbohydrate interactions by O→S/Se replacement at the glycosidic linkage, through the use of the unnatural aa *S*-(α-d-GalNAc)-thiothreonine (*S*Thr*) and *Se*-(α-d-GalNAc)-selenothreonine (*Se*Thr*) improved binding towards a representative anti-MUC1 antibody relative to native antigens. Importantly, the mice antisera recognized cancer cells in biopsies of breast cancer patients with high selectivity (Compañón et al., 2019).

Cell-penetrating peptides can pass through biological barriers together with a cargo and subsequently enter the cytoplasm. MPG is a cell-penetrating peptide derived from the HIV-1 gp41 protein, that can be used to facilitate peptide transport into cells (Liu et al., 2019). Using an FDA-approved poly (lactide-*co*-glycolide) acid nanoparticles as antigen-delivery vehicles, the OVA antigen was chemically linked to MPG, and subsequently encapsulated in nanoparticles. Use of the MPG-OVA-loaded nanoparticles elevated significantly the release of OVA into the cytosol of dendritic cells and promoted the maturation and activation of these professional antigen presenting cells. Furthermore, an expansion of OVA-specific T-cells was observed, together with the generation of OVA-specific IgG antibodies and the proliferation of OVA-specific memory T cells in mice vaccinated with the MPG-OVA-loaded nanoparticles. Moreover, the treatment of EG7-OVA tumor-bearing mice with the MPG-OVA-loaded nanoparticles resulted in significantly suppressed tumor growth and prolonged survival periods compared to treatment with unmodified OVA-nanoparticles or free OVA (Lindqvist et al., 2009).

More recently, ligand peptides designed from the crystal structures of pMHC were used to target specific MHC-I molecules on the surface of specific cells. These APL versions of peptides that already display high affinity to a specific MHC allele renders it possible to target a cargo to cells that express the specific MHC by replacing a peptide residue (Sun et al., 2020). One of the TCR-binding peptide residue can be replaced with chemically modified lysine residue a lysine with an ε-amine group modified with functional molecules. The designed ligand peptides successfully bound to cells expressing the corresponding MHC-I molecules via exchange of peptides bound to MHC-I. The peptide could be used to transport a protein or a liposome to cells expressing the corresponding MHC-I (Sun et al., 2020).

## Concluding remarks

Our immune system discriminates self from non-self by examining the peptide register presented by HLA molecules on cell surfaces. Successful recognition of MHC-restricted pathogen-derived and/or cancer-associated epitopes by T cells and NK cells can induce immune responses that lead to efficient destruction of infected cells. More recent technological advances in *e.g.* mass-spectrometry, including hybrid fragmentation technologies, provide us today with an unprecedented capacity to identify specific MHC-I and MHC-II epitopes in both healthy and stressed/infected cells. This opens for the clear possibility to make use of these presented peptides to unravel the complexity of the presented antigen population, providing further molecular insights into how the immunopeptidome is a surface replica of the proteome, and why some specific epitopes are presented instead of others. The identified peptides are also used to assess immune responses towards specific diseases, including vaccination, but also to identify specific T cell populations that could be used for tailored therapies. Besides the conventional linear peptides that were initially identified, it is now clear that HLA molecules also present very large ensembles of post-translationally modified epitopes. Previous and ongoing studies provide a wealth of essential information on the chemical and structural modifications, including novel classes of noncontiguous spliced peptides. The aim of this review was to focus mainly on the information gathered from structural analyses of MHC-restricted PTM epitopes, and to try to describe how they add to the already vastly polymorphic antigenic landscape that is presented to the immune system.
